# Impact of Perioperative Prophylaxis With *Enterococcus* Activity on Risk of Surgical-Site Infection After Pancreas Transplantation

**DOI:** 10.1097/TXD.0000000000001496

**Published:** 2023-06-08

**Authors:** Zachary A. Yetmar, Molly McCord, Brian D. Lahr, Yogish C. Kudva, Maria Teresa Seville, Wendelyn Bosch, Adley Lemke, Nitin N. Katariya, Kunam S. Reddy, Dana K. Perry, Janna L. Huskey, Tambi Jarmi, Aleksandra Kukla, Patrick G. Dean, Stacy A. Bernard, Elena Beam

**Affiliations:** 1 Division of Public Health, Infectious Diseases, and Occupational Medicine, Department of Medicine, Mayo Clinic, Rochester, MN.; 2 Department of Pharmacy, Mayo Clinic, Rochester, MN.; 3 Division of Biomedical Statistics and Informatics, Department of Quantitative Health Sciences, Mayo Clinic, Rochester, MN.; 4 Division of Endocrinology, Diabetes, Metabolism and Nutrition, Department of Medicine, Mayo Clinic, Rochester, MN.; 5 Division of Infectious Diseases, Department of Medicine, Mayo Clinic, Phoenix, AZ.; 6 Division of Infectious Diseases, Department of Medicine, Mayo Clinic, Jacksonville, FL.; 7 Division of Transplantation Surgery, Department of Surgery, Mayo Clinic, Phoenix, AZ.; 8 Division of Transplantation Surgery, Department of Transplantation, Mayo Clinic, Jacksonville, FL.; 9 Division of Nephrology, Department of Medicine, Mayo Clinic, Phoenix, AZ.; 10 Division of Nephrology, Department of Medicine, Mayo Clinic, Jacksonville, FL.; 11 Division of Nephrology and Hypertension, Department of Medicine, Mayo Clinic, Rochester, MN.; 12 Division of Transplantation Surgery, Department of Surgery, Mayo Clinic, Rochester, MN.

## Abstract

Surgical-site infection (SSI) is the most common early infectious complication after pancreas transplantation (PT). Although SSI has been shown to worsen outcomes, little data exist to guide optimal choices in perioperative prophylaxis.

**Methods.:**

We performed a retrospective cohort study of PT recipients from 2010–2020 to examine the effect of perioperative antibiotic prophylaxis with *Enterococcus* coverage. *Enterococcus* coverage included antibiotics that would be active for penicillin-susceptible *Enterococcus* isolates. The primary outcome was SSI within 30 d of transplantation, and secondary outcomes were *Clostridioides difficile* infection (CDI) and a composite of pancreas allograft failure or death. Outcomes were analyzed by multivariable Cox regression.

**Results.:**

Of 477 PT recipients, 217 (45.5%) received perioperative prophylaxis with *Enterococcus* coverage. Eighty-seven recipients (18.2%) developed an SSI after a median of 15 d from transplantation. In multivariable Cox regression analysis, perioperative *Enterococcus* prophylaxis was associated with reduced risk of SSI (hazard ratio [HR] 0.58; 95% confidence interval [CI], 0.35-0.96; *P* = 0.034). Anastomotic leak was also significantly associated with elevated risk of SSI (HR 13.95; 95% CI, 8.72-22.32; *P* < 0.001). Overall, 90-d CDI was 7.4%, with no difference between prophylaxis groups (*P* = 0.680). SSI was associated with pancreas allograft failure or death, even after adjusting for clinical factors (HR 1.94; 95% CI, 1.16-3.23; *P* = 0.011).

**Conclusions.:**

Perioperative prophylaxis with *Enterococcus* coverage was associated with reduced risk of 30-d SSI but did not seem to influence risk of 90-d CDI after PT. This difference may be because of the use of beta-lactam/beta-lactamase inhibitor combinations, which provide better activity against enteric organisms such as *Enterococcus* and anaerobes compared with cephalosporin. Risk of SSI was also related to anastomotic leak from surgery, and SSI itself was associated with subsequent risk of a poor outcome. Measures to mitigate or prevent early complications are warranted.

## Graphical abstract

**Figure:**
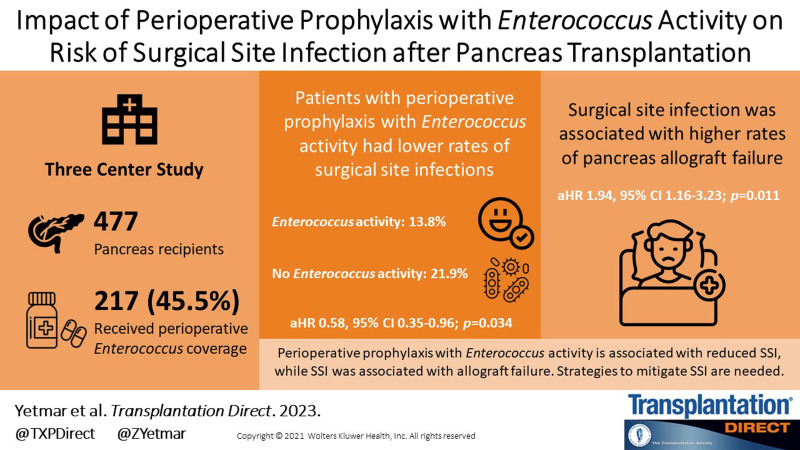

Approximately 1000 pancreas transplant (PT) procedures are performed annually in the United States. Nearly 10% of pancreas allografts fail within 90 d of transplantation, likely at least in part related to high rates of clinically significant infections following transplantation.^1,2^ Surgical-site infection (SSI) is the most common infection in the early posttransplant period, with an incidence estimated as high as 45%.^3-5^ Early infectious complications, and specifically SSI, have been associated with poor pancreatic allograft outcomes, with SSI recently being associated with a 17-fold increase in odds of pancreas allograft loss within 3 mo of transplantation.^5^ Intra-abdominal fungal infection, a subset of SSIs that typically involves the pancreas allograft, has also been associated with worsened pancreas allograft outcomes, including severe allograft dysfunction and allograft failure.^6,7^

Antibiotic prophylaxis is routinely used in the peritransplant period to prevent SSI. However, little data exist to guide the choice of antibiotic agents. One clinical trial randomly assigned PT recipients to receive either vancomycin with gentamicin or cefazolin with gentamicin.^8^ Although no difference was seen between regimens, only 24 PT recipients were enrolled, limiting the ability to detect a clinically meaningful difference in SSI rate. Other observational trials have typically used uniform perioperative prophylaxis^4,5^ or had an insufficient sample size to detect meaningful differences in outcomes.^9^ Furthermore, studies in nontransplant surgical populations, including neurologic, cardiovascular, and abdominal surgeries, have shown broad-spectrum prophylaxis to be associated with higher rates of *Clostridioides difficile* infection (CDI) without improving SSI rates.^10-13^ Additionally, CDI has been associated with subsequent allograft loss in solid organ transplant recipients overall.^14^

The lack of data regarding perioperative prophylaxis has led to inconsistent recommendations from leading professional societies for perioperative prophylaxis in PT. The guidelines from the American Society of Health-System Pharmacists, Infectious Diseases Society of America, Surgical Infection Society, and Society for Healthcare Epidemiology of America recommend PT recipients to receive perioperative prophylaxis with cefazolin.^15^ By contrast, the American Society of Transplantation guidelines recommend ampicillin–sulbactam.^16^

In this report, we aim to compare rates of SSI based on perioperative antibiotic prophylaxis regimen in PT recipients. Furthermore, we secondarily aim to analyze novel risk factors for SSI in the PT population and assess the impact of SSI on posttransplantation outcomes.

## MATERIALS AND METHODS

### Study Design and Setting

We performed a multicenter, retrospective cohort study of adult patients who underwent PT (including PT alone, pancreas-after-kidney transplantation, or simultaneous pancreas-kidney [SPK] transplantation) at our transplant centers in Arizona, Florida, and Minnesota from January 2010 through December 2020. In terms of perioperative antibiotic prophylaxis, the sites primarily used either ampicillin–sulbactam or piperacillin–tazobactam from 2010 to 2016. The choice of prophylaxis in this period was site-specific rather than risk specific. In 2017, all sites transitioned prophylaxis primarily to ceftriaxone or cefotaxime. Patients with reported beta-lactam allergies received a combination of vancomycin and aztreonam throughout the study period. Perioperative prophylaxis was routinely administered for 48 h postoperatively. All patients received universal fluconazole prophylaxis for 28 d posttransplant, dosed 200 mg once daily, which is extended to 1 y at the Arizona campus as *Coccidioides* prophylaxis.

These centers otherwise used common transplantation prophylaxis and immunosuppression protocols throughout the study period. Antimicrobial prophylaxis included trimethoprim–sulfamethoxazole for 6 mo and antiviral prophylaxis with valganciclovir for 3 to 6 mo or acyclovir for 30 d posttransplant, depending on donor and recipient cytomegalovirus serostatus. All pancreas allografts had enteric exocrine drainage. The duodenum was not opened before performing the anastomosis, and no selective bowel decontamination protocol was used. The anastomosis was double-layered handsewn using an absorbable suture for the mucosal layer and a permanent suture for the seromuscular layer. Induction immunosuppression was primarily with alemtuzumab or antithymocyte globulin. Standard posttransplant immunosuppression included tacrolimus, mycophenolate mofetil, and perioperative methylprednisolone followed by a prednisone taper. Select SPK recipients at low risk for allograft rejection received alemtuzumab induction immunosuppression followed by a rapid prednisone taper over 5 d with subsequent corticosteroid avoidance. Our institutional review board reviewed the study protocol and granted it an exempt status (#21-007258).

### Inclusion and Exclusion Criteria

PT recipients were identified from our internal transplant center registry and those transplanted between 2010 and 2020 were assessed for inclusion. Inclusion criteria were age ≥18 y at transplantation and receipt of an SPK, PT alone, or pancreas-after-kidney transplant. Exclusion criteria were pancreas allograft failure within 7 d of transplantation or lack of research authorization per state statute. Those with allograft failure within 7 d of transplantation were excluded because allograft failure in this early posttransplant period is most likely related to early pancreas allograft thrombosis. If patients underwent multiple PT procedures during the study period, the first transplant was included. Data from included patients were manually extracted from the electronic medical record. Abstracted data included demographics, pretransplant characteristics, peritransplant characteristics, and outcomes. Study data were collected and managed using REDCap electronic data capture tools hosted at the Mayo Clinic.^17,18^

The primary outcome was diagnosis of SSI within 30 d of transplantation. Secondary outcomes included CDI within 90 d of transplantation and long-term end points for pancreas allograft failure, death, and the composite of these 2. SSI was defined according to the Centers for Disease Control and Prevention SSI criteria with subcategorization as superficial incision, deep incisional, and organ/space SSI.^19^ CDI was defined as a positive stool *C difficile* polymerase chain reaction or toxin assay with concomitant diarrhea. Pancreas allograft failure was defined as a requirement of ≥0.5 units/kg/d of insulin for >90 d, relisting for PT, or undergoing allograft pancreatectomy. Perioperative antibiotic prophylaxis was categorized into 2 groups based on *Enterococcus* coverage. Prophylaxis agents with *Enterococcus* coverage included ampicillin–sulbactam, piperacillin–tazobactam, vancomycin-containing regimens, and levofloxacin-containing regimens. Agents without *Enterococcus* coverage included cephalosporin, namely cefazolin, ceftriaxone, cefotaxime, and cefepime. To better understand if *Enterococcus*-active prophylaxis was affected by use of uncommon regimens, we also defined a 3-level grouping variable for perioperative prophylaxis in which regimens with *Enterococcus* coverage were further categorized as typical (either ampicillin–sulbactam or piperacillin–tazobactam) or atypical (all others). Multidrug-resistant organisms (MDROs) included methicillin-resistant *Staphylococcus aureus* (MRSA), vancomycin-resistant *Enterococcus* (VRE), and multidrug-resistant Gram-negative bacilli. Multidrug-resistant Gram-negative bacilli included those resistant to at least 1 antibiotic in at least 3 different antibiotic classes. Ceftriaxone nonsusceptibility was used as a surrogate for extended-spectrum beta-lactamase–producing Gram-negative bacilli.

### Statistical Analysis

Descriptive statistics were generated with median and interquartile range (IQR) for continuous variables and number and percentage for discrete variables. The nonparametric loess smoother was used to graphically depict trends in the use of perioperative *Enterococcus* prophylaxis and the rate of 30-d SSI over the study period. For analyzing posttransplant outcomes, time-to-event analyses were conducted to account for patients lost to a >30-d follow-up and to properly incorporate time-dependent risk factors. For the purposes of this study, a poor long-term outcome was defined by a composite end point of pancreas allograft failure or death. For each outcome, we estimated the patients’ cumulative risk over time with Kaplan-Meier estimates.

A multivariable Cox proportional hazards regression model was used to assess the risk of 30-d SSI according to perioperative prophylaxis group (with or without *Enterococcus* coverage) and 9 covariates chosen a priori based on existing published research. We used the Anderson-Gill counting process, a formulation that allows for time-dependent covariates in the Cox model, to assess the intervening effects of pancreas graft thrombosis, acute allograft rejection, and reoperation. Time-fixed covariates included the antibiotic prophylaxis used, anastomotic leak, type of PT procedure, operative duration, alemtuzumab induction, pretransplant MDRO status, and hemoglobin A1c level. To avoid collinearity, operative times were standardized to the type of PT such that the transformed values for both types (SPK versus non-SPK transplant) had mean 0 and variance 1. We also repeated this Cox analysis using the 3-level perioperative prophylaxis variable with *Enterococcus*-active regimens further categorized as typical or atypical. Results are reported as hazard ratios (HRs) with 95% confidence intervals (CIs), except for 3-level prophylaxis categorization, which are presented with 97.5% CIs to adjust for multiple comparisons.

In a separate time-dependent Cox analysis, the relationship between the composite end point (time until pancreas allograft failure or death) and an intervening, time-dependent SSI up to day 30 was described by a covariate-adjusted model. Prespecified covariates included thrombosis and acute allograft rejection as additional time-dependent variables and age, sex, type of PT, and antibiotic prophylaxis as fixed variables. All Cox models were stratified by the Mayo site of transplantation to adjust for variation across these 3 settings. These analyses were done using R version 4.0.3 (R Foundation for Statistical Computing, Vienna, Austria).

## RESULTS

### Cohort Characteristics

During the study period, 504 adult patients underwent PT, of whom 27 (5.4%) were excluded because of pancreas allograft loss within 7 d of transplantation. The remaining 477 patients were included in these analyses. The median age of these transplant recipients was 44.2 y (IQR, 36.4–52.0), and 54.5% were male. The majority of these PT procedures were SPK (73.6%), most commonly for type 1 diabetes (87.6%). Of the SPK recipients, 70 (19.9%) were preemptive kidney transplants. Forty recipients (8.4%) had a known history of MDRO colonization before transplantation, most commonly MRSA (N = 32; 80%). Three patients had a history of both MRSA and VRE colonization. Baseline characteristics are further detailed in Table 1.

**TABLE 1.** Baseline characteristics

Variable	**Enterococcus coverage (N = 217**)	**No Enterococcus coverage (N = 260**)	**Overall (N = 477**)
**Pretransplant characteristics**			
Age, y, median (IQR)	45.0 (37.3–52.5)	43.8 (36.2–51.2)	44.2 (36.4–52.0)
Sex			
Female	100 (46.1)	117 (45.0)	217 (45.5)
Male	117 (53.9)	143 (55.0)	260 (54.5)
Race			
Asian	2 (0.9)	5 (1.9)	7 (1.5)
Black or African American	9 (4.1)	39 (15.0)	48 (10.1)
Indigenous American or Alaskan Native	1 (0.5)	3 (1.2)	4 (0.8)
Native Hawaiian or Other Pacific Islander	1 (0.5)	2 (0.8)	3 (0.6)
White	199 (91.7)	199 (76.5)	398 (83.4)
Other	5 (2.3)	12 (4.6)	17 (3.6)
BMI, kg/m^2^, median (IQR)	25.2 (22.6–28.5)	26.3 (24.0–28.7)	25.7 (23.3–28.6)
Pretransplant hemoglobin A1c, median (IQR)	8.0 (7.1-9.4)	7.7 (6.8-8.7)	7.9 (6.9-8.9)
History of CDI before transplantation	9 (4.1)	7 (2.7)	16 (3.4)
MDRO colonization before transplantation	18 (8.3)	22 (8.5)	40 (8.4)
Gram-negative bacilli	0 (0.0)	1 (0.4)	1 (0.2)
MRSA	13 (6.0)	19 (7.3)	32 (6.7)
VRE	7 (3.2)	3 (1.2)	10 (2.1)
Pretransplant abdominal surgery	106 (48.8)	108 (41.5)	214 (44.9)
Hospitalization within 30 d before transplantation	2 (0.9)	1 (0.4)	3 (0.6)
Chronic heart failure	3 (1.4)	7 (2.7)	10 (2.1)
Coronary artery disease	33 (15.2)	19 (7.3)	52 (10.9)
Hypertension	173 (79.7)	222 (85.4)	395 (82.8)
Reported beta-lactam allergy	61 (28.1)	8 (3.1)	69 (14.5)
Transplant characteristics			
PT type			
PT alone	42 (19.4)	36 (13.8)	78 (16.4)
Pancreas after kidney	31 (14.3)	17 (6.5)	48 (10.1)
SPK	144 (66.4)	207 (79.6)	351 (73.6)
PT indication			
Type 1 diabetes	208 (95.9)	210 (80.8)	418 (87.6)
Type 2 diabetes	8 (3.7)	48 (18.5)	56 (11.7)
Pancreatectomy	1 (0.5)	2 (0.8)	3 (0.6)
Redo pancreas	20 (9.2)	12 (4.6)	32 (6.7)
Kidney transplant indication^*a*^			
Diabetic nephropathy	141 (97.9)	195 (94.2)	336 (95.7)
Hypertensive nephrosclerosis	2 (1.4)	6 (2.9)	8 (2.3)
Other	1 (0.7)	6 (2.9)	7 (2.0)
Preemptive kidney transplantation^*a*^	40 (27.8)	30 (14.5)	70 (19.9)
Pretransplant peritoneal dialysis^*b*^	26 (25.0)	64 (36.2)	90 (32.0)
Operative duration, min, median (IQR)	245 (184–324)	243 (207–288)	245 (199–302)
Induction immunosuppression			
Alemtuzumab	71 (32.7)	149 (57.3)	220 (46.1)
Antithymocyte globulin	146 (67.3)	105 (40.4)	251 (52.6)
Basiliximab	0 (0.0)	6 (2.3)	6 (1.3)
Perioperative antibiotic choice			
Ampicillin–sulbactam	47 (21.7)	0 (0.0)	47 (9.9)
Cefazolin	0 (0.0)	5 (1.9)	5 (1.0)
Cefepime	0 (0.0)	1 (0.4)	1 (0.2)
Cefotaxime	0 (0.0)	108 (41.5)	108 (22.6)
Ceftriaxone	0 (0.0)	146 (56.2)	146 (30.6)
Levofloxacin + metronidazole	16 (7.4)	0 (0.0)	16 (3.4)
Piperacillin–tazobactam	114 (52.5)	0 (0.0)	114 (23.9)
Vancomycin	1 (0.5)	0 (0.0)	1 (0.2)
Vancomycin + aztreonam	34 (15.7)	0 (0.0)	34 (7.1)
Vancomycin + levofloxacin	3 (1.4)	0 (0.0)	3 (0.6)
Vancomycin + piperacillin–tazobactam	2 (0.9)	0 (0.0)	2 (0.4)
Anastomotic leak	16 (7.4)	20 (7.7)	36 (7.5)

Data are presented as n (%) unless otherwise specified.

^*a*^These data are out of 351 SPK transplant recipients.

^*b*^These data are out of 281 SPK transplant recipients who required pretransplant dialysis.

BMI, body mass index; CDI, *Clostridioides difficile* infection; IQR, interquartile range; MDRO, multidrug-resistant organism; MRSA, methicillin-resistant *Staphylococcus aureus*; PT, pancreas transplant; SPK, simultaneous pancreas-kidney; VRE, vancomycin-resistant *Enterococcus*.

Just over half of the cohort received perioperative prophylaxis without *Enterococcus* activity (N = 260; 54.5%). These were limited to cephalosporin antibiotics, almost entirely ceftriaxone or cefotaxime. Of the remaining patients (N = 216; 45.5%) who received prophylaxis with *Enterococcus* activity, three-fourths (N = 163; 75.1%) received typical agents, either with piperacillin–tazobactam (N = 116) or ampicillin–sulbactam (N = 47); the remainder (N = 54; 24.9%) received atypical regimens (most commonly vancomycin with aztreonam [N = 34]). No patients received VRE-active prophylaxis and the 1 patient with pretransplant multidrug-resistant Gram-negative colonization received ceftriaxone. Those who received *Enterococcus*-active perioperative prophylaxis were more likely to be of white race (91.7% versus 76.5%), have a reported beta-lactam allergy (28.1% versus 3.1%), and receive antithymocyte globulin induction immunosuppression (67.3% versus 40.4%). Those who did not receive *Enterococcus*-active prophylaxis had a higher proportion of patients with SPK transplantation (79.6% versus 66.4%) and indicated for PT by type 2 diabetes (18.5% versus 3.7%). Nearly all patients (N = 474; 99.4%) received posttransplant antifungal prophylaxis with fluconazole for a median of 32.0 (IQR, 30.0–360.5) d, with 86.4% receiving at least 30 d of prophylaxis.

### Surgical-Site Infection

After 30 d of posttransplant follow-up, 87 (18.2%) developed SSI (Table 2). These were predominantly organ/space infections (80.5%) and were diagnosed with a median of 15 d from transplantation (IQR, 10.5–21). Of those who developed SSI, 22 either did not have cultures obtained or cultures were without growth, whereas 7 had growth of >4 unique enteric organisms and the microbiology was not identified further. From the remaining 58 patients with evaluable microbiology, the most common organisms were coagulase-negative *Staphylococcus* (46.6%), *Candida* species (29.3%), *Enterococcus faecium* (20.7%), and *Streptococcus* species (20.7%). The most common *Candida* species was *C albicans* (8; 47.1%) followed by *C glabrata* (5; 29.4%), *C krusei* (3; 17.6%), and *C parapsilosis* (1; 5.9%). Fifteen (78.9%) of those with *Candida* had polymicrobial infections. Sixteen patients had SSI with an MDRO (27.6%). This was most commonly because of VRE (62.5%) and extended-spectrum beta-lactamase–producing Gram-negative bacilli (31.3%). There were no apparent differences in microbiology between those who did and did not receive *Enterococcus*-active perioperative prophylaxis (Table 3).

**TABLE 2.** Short- and long-term outcomes

Variable	**Overall (N = 477**)
Hospital length of stay, d, median (IQR)	7.0 (5.0–9.0)
30-d SSI	87 (18.2)
Superficial incisional	11 (12.6)
Deep incisional	6 (6.9)
Organ/space	70 (80.5)
30-d pancreas allograft thrombosis	34 (7.1)
30-d reoperation	71 (14.9)
30-d acute rejection^*a*^	25 (5.2)
CDI	
Day = 30	27 (5.7)
Day = 60	31 (6.5)
Day = 90	35 (7.4)
Death-censored pancreas allograft failure	
Month = 3	10 (2.1)
Month = 6	13 (2.7)
Month = 12	22 (4.7)
Month = 36	39 (9.3)
Month = 60	53 (14.7)
Mortality	
Month = 3	2 (0.4)
Month = 6	4 (0.8)
Month = 12	10 (2.1)
Month = 36	18 (4.2)
Month = 60	32 (9.5)
Composite of pancreas allograft failure or death	
Month = 3	11 (2.3)
Month = 6	15 (3.1)
Month = 12	29 (6.1)
Month = 36	49 (11.5)
Month = 60	70 (19.3)

Data are n (%) unless otherwise specified.

Cumulative incidence of CDI, death-censored allograft failure, mortality, and the composite of allograft failure or death are based on inversed Kaplan-Meier probabilities. Pancreas allograft failure was defined as a requirement of ≥0.5 units/kg/d of insulin for >90 d, relisting for PT, or allograft pancreatectomy; patient mortality is defined as death from any cause regardless of allograft function.

^*a*^30-d acute rejection included 19 with acute cellular rejection of the pancreas allograft, 2 with acute cellular and antibody-mediated rejection of the pancreas allograft, 2 with acute cellular rejection of the kidney allograft, and 2 with antibody-mediated rejection of the kidney allograft.

CDI, *Clostridioides difficile* infection; IQR, interquartile range; PT, pancreas transplantation; SSI, surgical-site infection.

**TABLE 3.** Microbiology of 58 SSI cases with identified organisms

Organism	**Enterococcus coverage (N = 19**)	**No Enterococcus coverage (N = 39**)	**Overall (N = 58**)
*Staphylococcus aureus*	1 (5.3%)	1 (2.6%)	2 (3.4%)
Coagulase-negative *Staphylococcus*	10 (52.6%)	17 (43.6%)	27 (46.6%)
*Streptococcus* spp.	3 (15.8%)	9 (23.1%)	12 (20.7%)
*Enterococcus faecalis*	4 (21.1%)	5 (12.8%)	9 (15.5%)
*Enterococcus faecium*	4 (21.1%)	8 (20.5%)	12 (20.7%)
*Enterococcus gallinarium*	1 (5.3%)	1 (2.6%)	2 (3.4%)
*Enterococcus casseliflavus*	0 (0.0%)	1 (2.6%)	1 (1.7%)
*Pseudomonas aeruginosa*	1 (5.3%)	3 (7.7%)	4 (6.9%)
*Escherichia coli*	2 (10.5%)	6 (15.4%)	8 (13.8%)
*Klebsiella* spp.	6 (31.6%)	2 (5.1%)	8 (13.8%)
*Enterobacter* spp.	0 (0.0%)	1 (2.6%)	1 (1.7%)
*Serratia* spp.	0 (0.0%)	1 (2.6%)	1 (1.7%)
*Citrobacter* spp.	0 (0.0%)	1 (2.6%)	1 (1.7%)
*Candida* spp.	4 (21.1%)	13 (33.3%)	17 (29.3%)
*Bacteroides* spp.	0 (0.0%)	5 (12.8%)	5 (8.6%)
Other	5 (26.3%)	7 (17.9%)	12 (20.7%)
Polymicrobial	11 (57.9%)	23 (59.0%)	34 (58.6%)
MDRO	4 (21.1%)	12 (30.8%)	16 (27.6%)
MRSA	0 (0.0%)	1 (8.3%)	1 (6.2%)
VRE	2 (50.0%)	8 (66.7%)	10 (62.5%)
ESBL Gram-negative bacillus	2 (50.0%)	3 (25.0%)	5 (31.2%)
Carbapenem resistant *Enterobacterales*	2 (50.0%)	0 (0.0%)	2 (12.5%)

Other organisms included *Lactobacillus* spp. (3), *Actinomyces odontolyticus*, *Anaerococcus*, *Campylobacter curvus*, *Clostridium clostridioforme*, *Clostridium perfringens*, *Finegoldia magna*, *Fusobacterium*, *Haemophilus parainfluenzae*, *Micrococcus luteus*, *Prevotella*, *Proteus mirabilis*, *Rothia mucilaginosa*, and *Veillonella dispar*. Note many infections sometimes involved >1 organism, resulting in column totals >100%.

ESBL, extended-spectrum beta-lactamase; MDRO, multidrug-resistant organism; MRSA, methicillin-resistant *Staphylococcus aureus*; spp., species; SSI, surgical-site infection; VRE, vancomycin-resistant *Enterococcus*.

**FIGURE 1.**
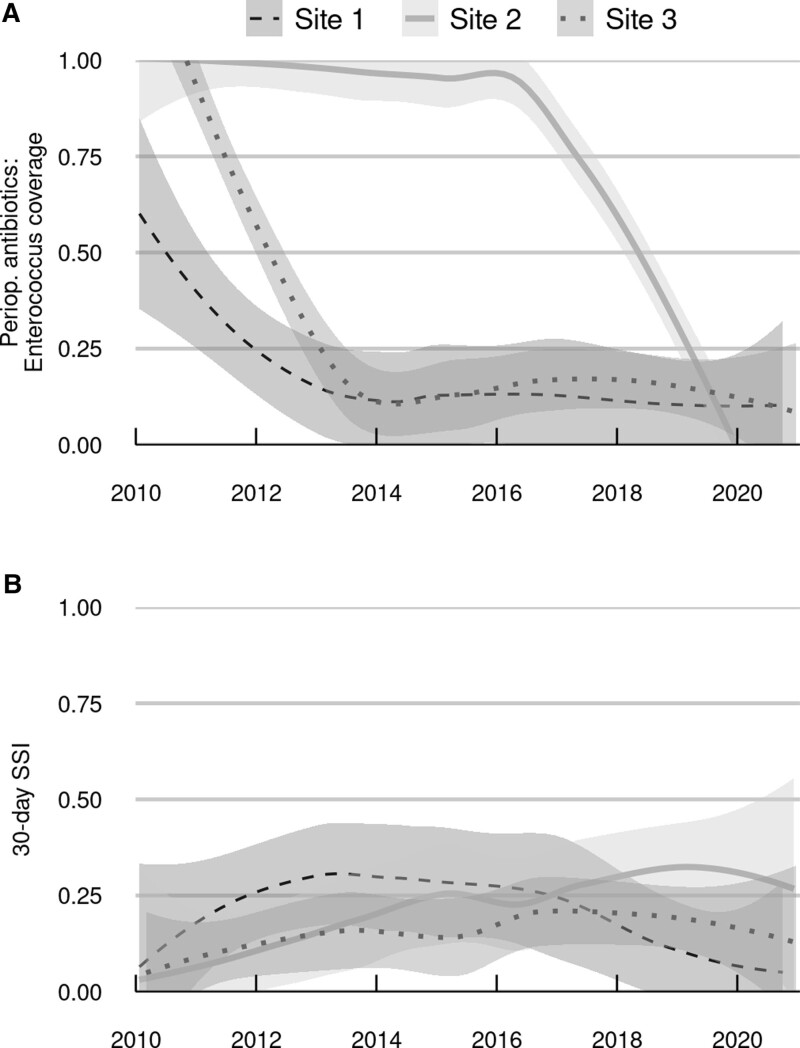
Curves showing smoothed trends in the use of perioperative prophylaxis with *Enterococcus* activity (A) and rates of SSI (B) over the study period, both stratified by study site. SSI, surgical-site infection.

*Enterococcus*-active prophylaxis use was more prominent early in the study period before institutional antimicrobial prophylaxis alignment in 2017, particularly at site 2 (Figure 1A). Of the 3 sites, only site 2 showed a significant increasing trend in the rate of 30-d SSI over time (*P = *0.007; Figure 1B). The prophylaxis group without *Enterococcus* coverage developed 57 (21.9%) SSIs, whereas the *Enterococcus*-active prophylaxis group had 30 (13.8%) SSIs. In multivariable analysis (Table 4), the use of prophylaxis with *Enterococcus* coverage was associated with a reduced risk of 30-d SSI (HR 0.58; 95% CI, 0.35-0.96; *P* = 0.034). Of the other 9 variables in the model, only anastomotic leak was significantly associated with SSI (HR 13.95; 95% CI, 8.72-22.32; *P* < 0.001). When this Cox analysis was repeated with *Enterococcus*-active regimens further classified as typical or atypical, the overall association between this 3-level variable and SSI was not statistically significant (*P* = 0.085); specifically, there was a modest but nonsignificant reduction in risk for both typical (HR 0.52; 97.5% CI, 0.26-1.05) and atypical (HR 0.69; 97.5% CI, 0.30-1.56) *Enterococcus* regimens relative to prophylaxis without *Enterococcus* coverage.

**TABLE 4.** Association of clinical factors with 30-d SSI

Variable	HR **(95% CI**)	*P*
Perioperative prophylaxis with *Enterococcus* coverage	0.58 (0.35-0.96)	0.034
Pancreas graft thrombosis	0.95 (0.35-2.58)	0.924
Anastomotic leak	13.95 (8.72-22.32)	<0.001
SPKtransplant	0.70 (0.40-1.22)	0.203
Pretransplant MDRO colonization	1.28 (0.64-2.59)	0.487
Pretransplant hemoglobin A1c	1.02 (0.78-1.33)	0.908
Alemtuzumab induction	1.22 (0.67-2.20)	0.520
Standardized operative duration	1.01 (0.74-1.38)	0.934
Reoperation	1.03 (0.45-2.31)	0.952
Acute rejection	1.88 (0.55-6.42)	0.312

CI, confidence interval; HR, hazard ratio; MDRO, multidrug-resistant organism; SPK, simultaneous pancreas-kidney; SSI, surgical-site infection.

### Secondary outcomes

At 90-d follow-up, the cumulative incidence of CDI was 8.9% (23/260) in the group provided no *Enterococcus* coverage and 5.5% (12/217) in the group with *Enterococcus* coverage; comparison of these rates in a Cox analysis stratified by site but uncontrolled for other factors showed no significant difference between groups (*P* = 0.680).

During a median follow-up of 4.6 y (IQR, 2.4–7.5), 55 patients died (5-y mortality = 9.5%), 63 patients experienced allograft loss (5-y risk = 14.7%), and 92 patients died or had allograft loss (5-y risk = 19.3%). Using a time-dependent multivariable Cox analysis, development of 30-d SSI was associated with subsequent risk of the composite end point for death or allograft failure, after adjusting for age, sex, PT type, *Enterococcus*-active perioperative prophylaxis, pancreas allograft thrombosis, and acute rejection (HR 1.94; 95% CI, 1.16-3.23; *P* = 0.011). None of these adjustment factors were significantly associated with the composite end point (*P* > 0.3).

## DISCUSSION

In this study, the use of *Enterococcus*-active perioperative prophylaxis was associated with a reduced risk of 30-d SSI following PT in analysis adjusted for 9 clinical variables. Other than the prophylaxis group, only anastomotic leak was independently associated with increased risk of SSI, whereas novel factors such as pretransplant MDRO colonization and pretransplant glycemic control were not significant predictors. However, SSIs, primarily consisting of organ/space infections, were very common in this cohort and were independently associated with the long-term composite end point of pancreas allograft failure or death.

Optimal perioperative prophylaxis for PT recipients has undergone little investigation. A prior randomized trial attempted to answer the question of optimal prophylaxis.^8^ However, this study included mostly kidney transplant recipients and primarily evaluated expanded Gram-positive coverage through the use of vancomycin over cefazolin, which has been associated with more poor outcomes in other populations.^15,20^ All other studies of SSI in the PT population are retrospective, and most did not have a change in prophylaxis protocols and were thus unable to evaluate impact of prophylaxis.

The data presented here suggest that prophylaxis with *Enterococcus* activity provides better SSI protection over other agents, which primarily consist of ceftriaxone or cefotaxime. One important consideration is that these *Enterococcus*-active prophylaxis regimens were primarily composed of beta-lactam/beta-lactamase inhibitor combinations, compared to cephalosporin. These combinations usually also include antianaerobic activity and broad activity against enteric organisms. Indeed, 183 of 217 patients (84.3%) who received *Enterococcus*-active prophylaxis also included anaerobic coverage through beta-lactam/beta-lactamase inhibitor combinations or metronidazole. Furthermore, when *Enterococcus*-active agents were subclassified as typical or atypical, a comparison of typical regimens with beta-lactam/beta-lactamase inhibitor combinations versus cephalosporin yielded a similar (albeit statistically nonsignificant) reduction in risk of SSI as the primary analysis, whereas the effect of atypical regimens was somewhat attenuated. As such, our data support the recommendations from the American Society of Transplantation, favoring ampicillin–sulbactam as the preferred prophylaxis regimen among patients undergoing PT.^16^ Use of beta-lactam/beta-lactamase inhibitor combinations, such as ampicillin–sulbactam, is preferred from an antimicrobial stewardship perspective and appears to provide more optimal SSI prophylaxis.

In contrast to studies of other surgical populations, broader prophylaxis (in this study, surrogated by *Enterococcus* coverage) was not significantly associated with development of CDI. Many of the previously studied surgical populations have much lower rates of overall and organ/space SSI than PT recipients.^21^ Patients with organ/space SSIs often receive prolonged courses of antibiotics, which would likely lead to more CDI than perioperative prophylaxis alone. Additionally, most of the agents used for perioperative prophylaxis in the PT population are targeted at enteric organisms rather than skin flora, which have higher risk for CDI.^22^ In total, efforts should be made to minimize antimicrobial exposure to reduce risk of CDI and colonization with drug-resistant organisms such as VRE and MDR-Gram-negative organisms. Although the use of *Enterococcus*-active prophylaxis regimens, primarily beta-lactam/beta-lactamase inhibitor combinations, did not appear to influence rates of CDI, optimization of perioperative prophylaxis can potentially prevent SSIs and indirectly prevent posttransplant CDI.

Additionally, these data implicate anastomotic leak to be impactful in the subsequent development of SSI. Similarly, a recent analysis of PT recipients with anastomotic leak suggested a similar association with intra-abdominal infection.^23^ Although this recent study found anastomotic leak to be associated with pancreas allograft failure, our study suggests that SSI also contributes to this risk. Combined, these data call for a need for strategies to prevent anastomotic leak or minimize its consequences. In the quoted study, patients who underwent salvage operation for anastomotic leak had overall good outcomes, although intra-abdominal abscess was associated with a decreased chance of successful salvage.^23^ It stands to reason that earlier intervention before abscess formation may provide the best chance for allograft salvage, for which methods for early leak identification would be needed.

Previously described risk factors for SSI after PT have included reoperation, prolonged operative time, longer ischemic time, pancreas allograft rejection, allograft thrombosis, and SPK transplantation.^5,16,24,25^ However, our analysis found several of these to not be associated with SSI after adjustment for other factors. Some of these contrasting findings may be because of differences in populations because many studies solely included SPK recipients. Furthermore, many of these factors likely represent more complicated operations, such as longer operative time, and may also be associated with anastomotic leak. Ultimately, anastomotic leak is likely one of the common pathways to organ/space SSI in this population, although factors like allograft thrombosis or prolonged operative time may contribute to the development of leaks.

We did evaluate 2 novel factors of pretransplant MDRO colonization and pretransplant glycemic control, as measured by hemoglobin A1c immediately before transplantation. Poor glycemic control has been associated with SSI in nontransplant population,^26-28^ although it was not a significant association in the present analysis. The PT population is inherently high risk for poor glycemic control, as this is typically the indication for transplantation, and it is possible that there is little differential effect from different degrees of suboptimal control. Although pretransplant MDRO colonization did not appear to predict posttransplant SSI, nearly all with pretransplant MDROs were colonized with MRSA. *Staphylococcus aureus*, and MRSA specifically, was very uncommon in the evaluated SSI microbiology, and this sample does not adequately address colonization with VRE or resistant Gram-negative bacilli. These resistant organisms also have different epidemiology, and individual patient factors should be considered.

The SSI microbiology is notable for its high prevalence of coagulase-negative *Staphylococcus*, *Enterococcus* species, and *Candida* species. Coagulase-negative staphylococci have frequently been described as a prominent organism in SSIs in PT recipients, with rates up to 52.7%.^5,29^ Interestingly, the rates of *Enterococcus* isolation were similar between those who did or did not receive *Enterococcus*-active prophylaxis. This may have been influenced by rates of penicillin and vancomycin resistance. However, the effect of *Enterococcus*-active prophylaxis regimens may not direclty prevent enterococcal infections but broad activity against enteric organisms from beta-lactam/beta-lactamase inhibitor combinations. Additionally, patients with anastomotic leaks or SSIs late in the first 30 d posttransplant may not be as affected by the spectrum of prophylaxis. Instead, early transplant-related complications may influence risk and microbiologic in these settings.

*Candida* was a predominant pathogen despite universal 28-d fluconazole prophylaxis. Notably, nearly half of the *Candida* species were *C glabrata* or *C krusei*, for which low-dose fluconazole would not be expected to provide effective prophylaxis. We did not have a comparator group without antifungal prophylaxis, and thus cannot assess its impact on the rates of SSI from fungal organisms. However, the high rate of *Candida* involvement questions the efficacy of this intervention. Instead, fluconazole prophylaxis in PT recipients may simply select for more resistant *Candida* species.

Our study identified an independent association of 30-d SSI with subsequent risk of pancreas allograft failure or death, although lower in magnitude than previous studies.^5^ Some of this may be because of adjustment for other potential confounding factors and exclusion of patients with early allograft failure. Additionally, differences between transplant protocols, such as induction immunosuppression, or patient populations may have influenced these results. Along with SSIs, anastomotic leak has been shown to be highly impactful in terms of pancreas allograft failure.^23^ These 2 factors have consistently been shown to be correlated with poor pancreas allograft outcomes.^5,23^ These data suggest that further efforts to prevent SSIs may lead to better allograft outcomes in this population. Efforts to intervene in anastomotic leaks may be able to prevent SSIs. Additionally, patients who undergo successful operative salvage for anastomotic leaks have similar long-term outcomes to those without posttransplant leaks. Further efforts to recognize and address anastomotic leaks early following PT may reduce rates of SSI and allograft failure.

This study has several limitations of note. It was conducted retrospectively and may be subject to intrinsic sources of bias. We excluded patients with graft failure within 7 d of transplantation because these are often because of early pancreas graft thrombosis; however, this likely excluded the most significant graft thromboses, which may have diluted an effect from graft thrombosis on SSI incidence. Our transplant program also does not systematically screen all transplant candidates for MDRO colonization, and data for this variable were based on clinical cultures and screening for other purposes. It is possible some patients were misclassified as having no MDRO colonization because of lack of screening. There is also a potential for a time effect confounding the relationship between spectrum of prophylaxis and SSI development, although there was little change in SSI incidence over the study period. There are also potential differences in effectiveness between specific antibiotics in the prophylaxis categories. Not all patients with SSI had evaluable microbiology, likely because of a lack of culture ascertainment with more superficial infections or receipt of antibiotics before collection. Although we did find an association between SSI and pancreas allograft failure or mortality, this relationship may be affected by unmeasured confounding from high-risk characteristics in certain transplant recipients. Finally, this study suggests that organ/space infection is the most common SSI depth in the PT population, although it is possible that some superficial and deep incisional SSIs were not diagnosed at the sites or adequately recorded in the medical record and thus missed in the data abstraction process.

This study found perioperative prophylaxis with *Enterococcus* activity, primarily beta-lactam/beta-lactamase inhibitor combinations, to be associated with a lower risk of 30-d risk of SSI in PT recipients. This finding is consistent with recommendations from the American Society of Transplantation,^16^ and the use of beta-lactam/beta-lactamase inhibitor combination antibiotics such as ampicillin–sulbactam appear to be preferred over cephalosporin prophylaxis. Additionally, anastomotic leak was highly associated with developing SSI, which itself was independently associated with long-term risk of pancreas allograft failure or death. Further studies, such as diagnostic testing to identify early anastomotic leaks and methods to optimally address leaks, are needed to prevent associated complications of SSI and pancreas allograft failure.

## ACKNOWLEDGMENTS

Preliminary results of this work were presented at American Transplant Congress 2022 (abstract #C136).

## Supplementary Material


