# Supplementary figures and images for: Impact of Perioperative Prophylaxis With Enterococcus Activity on Risk of Surgical-Site Infection After Pancreas Transplantation
Source: Transplant Direct. 2023 Jun 8;9(7):e1496. doi: 10.1097/TXD.0000000000001496 (PMC10256365; doi:10.1097/TXD.0000000000001496)

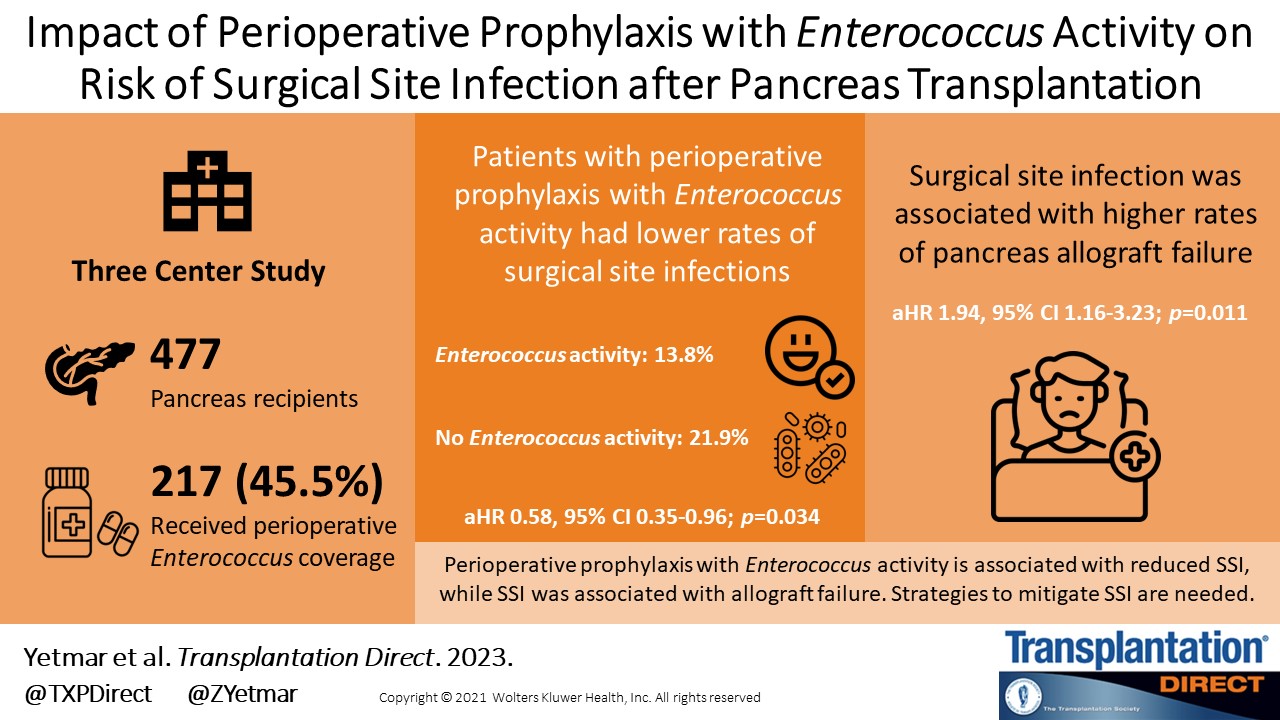

Supplement: Supplementary file 1 [file txd-9-e1496-s001.jpg]
